# Controlled Substance Agreements for Opioids in a Primary Care Practice

**DOI:** 10.1186/s40545-017-0119-5

**Published:** 2017-09-12

**Authors:** Lindsey M. Philpot, Priya Ramar, Muhamad Y. Elrashidi, Raphael Mwangi, Frederick North, Jon O. Ebbert

**Affiliations:** 1 Robert D. and Patricia E. Kern Mayo Clinic Center for the Science of Health Care Delivery, Mayo Clinic College of Medicine, 200 1st Street SW, Rochester, MN 55905 USA; 2 Primary Care Internal Medicine, Mayo Clinic College of Medicine, Rochester, MN USA

## Abstract

**Background:**

Opioids are widely prescribed for chronic non cancer pain (CNCP). Controlled substance agreements (CSAs) are intended to increase adherence and mitigate risk with opioid prescribing. We evaluated the﻿ demographic ﻿characteristics of a﻿nd opioid dosing for patients with CNCP enrolled in ﻿CSAs in a primary care practice.

**Methods:**

We conducted a retrospect﻿ive cohort study of﻿ 1066 patients enrolled in CSAs between May 9, 2013﻿ and August 15, 2016 for CNCP in a Midwest primary care practice.

**Results:**

Patients were prescribed an average of 40.8 (SD ± 57.0) morphine milligram equivalents per day (MME/day), and 21.5% of patients were receiving ≥50 MME/day and 9.7% were receiving ≥90 MME/day. Patients who were younger in age (≥ 65 vs. < 65 years, *P* < 0.0001), male gender (*P* = 0.0001), and used tobacco (*P* = 0.0002) received significantly higher MME/day. Patients with more co-morbidities (Charlson Comorbidity Index, CCI) received higher MME/day (CCI > 3 vs. CCI ≤ 3, *P* = 0.03), and reported higher average pain (CCI > 3 mean 5.8 [SD ± 2.1] vs. CCI ≤ 3 mean 5.3 [SD ± 2.0], *P* = 0.0011). Patients on an identified tapering plan (6.9%) had higher MME/day than patients not on a tapering plan (*P* = 0.0002).

**Conclusions:**

CSAs present an opportunity to engage patients taking higher doses of opioids in discussions about opioid safety, appropriate dosing and tapering. CSAs could be leveraged to develop a population health management approach to the care of patients with CNCP.

## Background

Pain is the one of the most common reasons that people seek medical care [1]. An estimated 14.6% of U.S. adults experience chronic (≥3 months) regional or widespread pain [2], and 25.3 million adults (11.2%) suffer chronic daily pain [3]. Up to one-third of patients in the primary care setting pain suffer from chronic non cancer pain (CNCP) [4]. Opioids are commonly prescribed for CNCP [5] in primary care despite their unproven long-term efficacy for this indication [6, 7].

Controlled substance agreements (CSAs) have been developed as a clinical risk mitigation strategy and are recommended by clinical practice guidelines [8, 9]. CSAs are documented agreements providing education and mutual consent between patients and providers informing patients of their responsibilities when using prescribed opioids [10]. CSAs have been associated with modest reductions in the misuse of prescribed opioids [11]. Despite their widespread use for patients receiving opioids, no consensus exists on the goals and compositions of CSAs [12].

Published studies have evaluated the content of CSAs [13], how frequently they are used [14–16], and how frequently enrolled patients abuse opioids [17]. Previous studies have described patient characteristics and indications for and types of opioids prescribed to patients with CNCP in primary care [4, 18, 19] and in specialty care pain clinics [20]. However, few published studies [19] have described the clinical characteristics of patients on CSAs for CNCP and the amount, type and dose of opioids they receive and the degree to which daily dosing exceeds recommendations in the recently released Centers for Disease Control and Prevention (CDC) clinical practice guideline [7]. Previous studies of patients on CSAs have not assessed the relationship between opioid dose and patient characteristics.

In the present study, we analyzed patients receiving opioids for CNCP enrolled in a CSA through a primary care practice in the Midwest United States. We report on the clinical characteristics of enrolled patients and the type and amount of opioids they received. We explore associations between total daily opioid doses received and demographics characteristics.

## Methods

### Study overview

We conducted a retrospective cohort study using patient data collected at CSA enrollment, administrative sources, and electronic health record (EHR) chart review and abstraction.

### Study setting

The study took place at the Mayo Clinic, a tertiary care academic medical center with a multispecialty primary care practice serving patients in Rochester, Minnesota and the surrounding area. This multispecialty primary care practice includes the divisions of internal medicine, family medicine, and pediatric/adolescent medicine which are situated within five distinct practice sites and provide care to approximately 152,000 patients.

### Study population

Individuals were included in our cohort if they were placed on a CSA for opioid therapy for CNCP between May 9, 2013 and August 15, 2016 within our primary care practice. The Mayo Institutional Review Board reviewed and approved this research. Patients were only included if they had provided research authorization.

### CSA enrollment

Our institutional guidelines recommend enrolling patients in a CSA if they are expected to be on a DEA Schedule II, III, or IV medication for ≥3 months. Enrollment is not expected for hospice, nursing home, palliative care, or group home patients. Clinicians can exercise discretion on enrollment if patients are receving less than ten pills per month. Upon CSA enrollment, nursing staff discuss CSA expectations with patients. Language on the CSA form includes direction on only having a single provider or health care team prescribe medications, safe medication storage, prohibitions on medication sharing and medication dose changes without clinician contact, urine drug testing requirements, follow-up appointment attendance, and requesting refills at least 1 week before renewal. Both the nursing staff member and patient sign the form which is scanned into the EHR. After CSA enrollment is completed, the Minnesota Prescription Drug Monitoring Program is queried.

### Data collection

#### CSA

Information collected at the time of CSA enrollment included patient demographics, the primary indication for chronic opioid therapy, and screening tests for depression and anxiety if the patient had a documented history of anxiety and/or depression. Patients with this history completed the Patient Health Questionnaire-9 Item Scale (PHQ-9) [21] to screen for depression and the Generalized Anxiety Disorder-7 Item Scale (GAD-7) [22] to screen for anxiety. Data were stored in a secured, intranet-based registry environment.

#### Administrative data

Data were extracted from administrative data feeds of patient provided information and billing data, and a data collection window of up to 1 year prior to program enrollment was used. Patient provided information included race, educational status, employment status, relationship status, and alcohol and/or tobacco use. This information is collected using a current visit information form completed yearly or as needed by patients at an outpatient clinic visit or inpatient hospitalization. Patient data is entered into discrete data fields upon completion and are extracted electronically. Due to decreased availability of these data elements for all patients across the study period, we provided the number of patients with this data available within all results tables. Administrative billing data 1 year prior to enrollment date were used in applying an institutional protocol to calculate age-weighted Charlson Comorbidity Index [23–25] (CCI) for each patient to serve as a measure of comorbidity burden.

#### EHR data

Chart abstraction was performed to determine details of opioid therapy at CSA initiation (opioid type, formulation, dose, and dose frequency), evidence of an opioid tapering plan, pain score (current pain, weekly average pain, weekly worst pain), and CSA status (active or terminated contract). Active status was defined as currently receiving an opioid prescription and terminated status was defined as opioid presciptions not currently being supplied. If the patient was taking more than one opioid at CSA initiation, the most potent opioid was listed first. If patients did not pick up their first prescription, they were not included in the cohort.

### Statistical analyses

Morphine milligram equivalents per day (MME/day) were calculated in order to allow for comparisons across opioid types. Total opioid dose per day was calculated by multiplying the amount of opioid per dose by the maximum prescribed doses per day. MME/day was calculated by multiplying total opioid dose per day by a morphine equivalent conversion factor [26, 27]. Bivariate analyses were performed to understand differences between populations by demographic characteristics, CCI, contract status, total and reported average pain, and MME/day.

Each variable was treated as continuous and checked for normality using histogram plots, measures of skewedness and kurtosis, and the Shapiro-Wilk Test for Normality. MME/day were not normally distributed and comparisons were made using the Wilcoxon Two Sample Test. Reported average pain was normally distributed and differences between groups was ascertained using a Two Sample t-Test. Pooled t-statistics are provided where the Folded F Equality of Variance estimates were equal (*p* > 0.05) and Satterthwaite t-statistics where variances were unequal (*p* ≤ 0.05). All data management and statistical analyses were performed using Statistical Analysis Software (SAS) Version 9.3 (Cary, North Carolina). In order to estimate the percentage of patients receiving opioid amounts above specific thresholds, we dichotomized opioid amounts above 50 MME/day and 90 MME/day.

## Results

### Demographics

We identified 1066 patients enrolled in a CSA with an average age of 63.6 years (standard deviation [SD] ± 15.1) of whom 65.7% were female (Table 1). More than one-half of patients had completed at least some college (54.9%). More than one-third of patients (37.3%) were retired, and 64.3% had a public payer as their primary insurer. Four percent of patients indicated a need to cut down on their alcohol consumption and 15.9% indicated that they use tobacco.

**Table 1:** Demographic Characteristics of 1066 Patients Enrolled in a Controlled Substance Agreement

Age (Mean (SD))	63.6 (15.1)
Gender (n (%))	
Female	700 (65.7)
Male	366 (34.3)
Race (n (%))	
Black	25 (2.3)
Other/Unknown	40 (3.8)
White	1001 (93.9)
Marital Status (n (%))	
Divorced	167 (15.7)
Married	607 (56.9)
Single	143 (13.4)
Widowed	149 (14.0)
What is the highest grade or level of school that you have completed? (n (%))	
High School or Less	260 (24.4)
Some College or 2 yr. Degree	351 (32.9)
4-year College Graduate	130 (12.2)
Graduate and Post Graduate Studies	105 (9.8)
Missing	220 (20.6)
What is your current employment status (check all that apply)? (n (%))	
Employed	237 (22.2)
Full Time Homemaker	25 (2.3)
Other	35 (3.3)
Retired	398 (37.3)
Self-Employed	29 (2.7)
Unemployed	43 (4.0)
Work Disabled	122 (11.4)
Missing	176 (16.5)
Insurance Information (n (%))	
Medicare	596 (55.9)
Other Government	90 (8.4)
Private	380 (35.7)
Felt the need to cut down on alcohol consumption (n (%))	
No	873 (81.9)
Yes	46 (4.3)
Missing	147 (13.8)
Current tobacco use (n (%))	
No	736 (69.0)
Yes	170 (15.9)
Missing	160 (15.0)

*SD* Standard Deviation

Significant differences were observed in MME/day by age (≥ 65 years mean 35.8 MME/day [SD ± 50.0] vs. < 65 years mean 45.0 MME/day [SD ± 62.0] Wilcoxon Two Sample Test, *t* Approximation, *P* < 0.0001), gender (females mean 35.2 MME/day [SD ± 42.4] vs. males mean 49.8 MME/day [SD ± 73.8]; Wilcoxon Two Sample Test, *t* Approximation, *P* = 0.0001), and tobacco use status (non tobacco user mean 39.5 MME/day [SD ± 61.1] vs. tobacco user mean 49.5 MME/day [SD ± 55.6]; Wilcoxon Two Sample Test, *t* Approximation, *P* = 0.0002). No significance differences in MME/day were observed between education (≤ high school vs. > high school, *P =* 0.20*)*, patient living arrangements (living alone vs. all other, *P* = 0.64) or between reporting the need to cut down on alcohol consumption compared those who did not (*P* = 0.27).

No significant differences were observed between average pain scores by age, gender, living arrangement, tobacco use status, education, or reported need to cut down on alcohol consumption.

### Opioids indication, pain scores and co-morbidity

Musculoskeletal pain (67.6%), chronic pain syndrome (14.6%), and neuropathy (8.5%) were the three leading indications among patients on opioid CSAs (Table 2). Patient reported current pain scores were a mean of 4.2 (SD ± 2.5) and worst pain scores were a mean of 7.6 (SD ± 2.1). Eight percent of patients had moderate to severe anxiety (GAD ≥10) and 11.4% had moderate to moderately severe depression (PHQ-9 ≥ 10). The overall population had an average age-weighted CCI of 3.9 (SD ± 3.1). Patients with higher CCI scores were taking significantly higher opioid doses (CCI > 3 mean 51.7 MME/day [SD ± 87.2] vs. CCI ≤ 3 mean 37.7 MME/day [SD ± 44.1] Wilcoxon Two Sample Test, *t* Approximation, *P* = 0.03). Patients with CCI ≤ 3 also reported lower average pain (CCI ≤ 3 mean 5.3 [SD ± 2.0] vs. CCI > 3 mean 5.8 [SD ± 2.1], Pooled Two Sample *t*-Test, *t =* 3.27, *P* = 0.0011).

**Table 2:** Indication for Opioids, Pain Scores and Co-Morbid Medical and Psychiatric Diagnoses (*N* = 1066)

Condition (n (%))	
Musculoskeletal pain	721 (67.6)
Chronic pain syndrome	156 (14.6)
Neuropathy	91 (8.5)
Headache/Migraine	45 (4.2)
Abdominal pain	11 (1.0)
Cutaneous/subcutaneous	10 (0.9)
Colorectal disease	8 (0.8)
Autoimmune disease	7 (0.7)
Nephrolithiasis	3 (0.3)
Sleep disorder	3 (0.3)
Autonomic dysfunction	2 (0.2)
Multiple sclerosis	2 (0.2)
Sarcoidosis	2 (0.2)
Syringomyelia	2 (0.2)
Angina	1 (0.1)
Dyspnea	1 (0.1)
Lymphedema	1 (0.1)
Age-Weighted Charlson Index (Mean (SD))	3.9 (3.1)
Pain (Mean (SD), *N* = 860)	
Current	4.2 (2.5)
Average	5.4 (2.1)
Worst	7.6 (2.1)
Moderate to Severe Anxiety Diagnosis (GAD-7 ≥ 10), N (%), *N* = 578	83 (7.8)
Moderate to Moderately Severe Depression Diagnosis (PHQ-9 ≥ 10), N (%), *N* = 785	121 (11.4)

*SD* Standard Deviation

### Opioids and contract status

Eighty-nine percent of patients had only one type of opioid prescribed (Table 3). Tramadol (54.5%) and Oxycodone (23.5%) were the most commonly prescribed opioids. Patients were on an average of 40.8 (± 57.0 SD) MME/day. A minority of patients (21.5%) were receiving ≥50 MME/day and 9.7% were receiving ≥90 MME/day.

**Table 3:** Opioid Prescriptions and Agreement Status

Number of Opioids Prescribed, N (%)	
1	948 (88.9)
2	111 (10.4)
3	7 (0.7)
Opioid Prescribed, N (%)	
Tramadol	581 (54.5)
Oxycodone	250 (23.5)
Hydrocodone	113 (10.6)
Codeine	52 (4.9)
Morphine	31 (2.9)
Hydromorphone	17 (1.6)
Fentanyl	9 (0.8)
Methadone	10 (0.9)
Other	3 (0.3)
Morphine Milligram Equivalents Per Day	
Mean (SD)	40.8 (57.0)
Median (Q1, Q3)	25.0 (15.0, 45.0)
Mode	20
Range	2.5–743.0
Medication change (Dose Change, New Opioid), N (%)	144 (13.5)
Tapering Plan, N (%)	74 (6.9)
Agreement Termination Reason, N (%),	
Admission to hospice/palliative care	12 (1.1)
Death	20 (1.9)
Contract violation or patient preference	126 (11.8)
Contract termination	32 (3.0)

*SD* Standard Deviation

Contracts had been terminated in 18% of patients by the end of the study period, 66% of which (*N* = 126) were discontinued for contract violation or patient preference. No significant differences were observed in MME/day or in pain scores between patients having a contract end during the study and those who did not (MME/day *P* = 0.26; average pain, Pooled *t*-Test *t* = 1.49, *P* = 0.37). Only 6.9% of patients were on an opioid tapering plan. MME/day were significantly higher among patients with an opioid tapering plan (mean 58.3 MME/day [SD ± 60.6] vs. mean 39.5 MME/day [SD ± 56.5]; Wilcoxon Two Sample Test, *t* Approximation, *P* = 0.0002), while reported average pain scores did not differ (Pooled *t*-Test, *t* = 0.3; *P* = 0.74).

## Discussion 

In our cohort of primary care patients on CSAs for CNCP in a Midwest primary care practice, we observed that patients who were younger in age, male gender, and used tobacco received higher MME/day. Patients with more co-morbidities and higher reported average pain were receiving higher MME/day. Patients who were on an identified tapering plan had higher MME/day than patients not on a tapering plan and 18% of the CSAs were discontinued by the end of the study period.

Compared to previous studies in the primary care population [4, 18, 19] our study sample was older [4, 19] with more medical comorbidity. The leading indication for opioid use in our population was musculoskeletal pain which is consistent with other studies of opioid prescribing in primary care [4, 18, 19]. Comorbid anxiety and depression were significantly higher in previous studies of CNCP patients in the primary care setting. In a study of 209 patients receiving opioids for CNCP, 36% had depression and 21% of women and 9% of men had anxiety [18]. In a study of 48 patients receiving opioids for CNCP, 54% had depression and 21% had anxiety [4]. However, these previous studies assessed lifetime depression through chart review while we assessed for current anxiety and depression using the PHQ-9 and GAD-7 at CSA enrollment. We observed a prevalence of moderate to severe anxiety of 7.8% and moderate to moderately severe depression of 11.4%. Depression is a risk factor for medical non adherence [28]. Patients with comorbid chronic pain and anxiety and depression are more likely to continue opioids [29] and to develop opioid use disorder [30]. Clinical assessment tools such as the Opioid Risk Assessment Tool (ORT) have been designed to assess the probability of a patient displaying aberrant behaviors when prescribed opioids for CNCP. However, screening tools such as the ORT are not routinely employed in clinical practice. The CDC guideline on opioid prescribing for chronic pain suggested that clinicians should not overestimate the ability of these tools to rule out risks from long-term opioid therapy [7]. A need exists for the development of effective screening tools to risk-stratify patients initiating CSAs. Such tools could allow clinical practices to engage patients at higher risk for opioid use disorders in counseling or more frequent follow-up and monitoring as opposed to a “one size fits all” strategy which may currently pervade clinical practice.

The daily prescribed opioid dose in our population (mean 40.8 MME/day) is comparable to a mean of 50–60 MME/day observed in a previous study of patients on long-term opioids for CNCP enrolled in two health plans serving over 1% of the U.S. population [26]. Our observed MME/day is lower than that observed in a study of 889 patients on opioids for CNCP in primary care with a mean of 92 MME/day [28]. The observed MME/day in our study is significantly lower than the median 180 MME/day observed in a Canadian study of patients attending a specialty chronic pain clinic for CNCP [20]. Our results also differ from these studies with respect to the most commonly prescribed opioids. Our patients most commonly received tramadol compared to hydrocodone [26], hydromorphone [20], and oxycodone [28]. Differences in the type of opioid prescribed to patients likely reflect regional practice patterns or health benefit design as evidenced by the differences in oxycodone prescribing for long-term opioid use between Kaiser Permanente (3%), Group Health Collaborative in Washington State (21%) [26], our Midwest population (23%), and five Wisconsin healthcare systems (50%) [28]. Available data suggests oral oxycodone has an elevated abuse liability profile compared to oral morphine and hydrocodone [31]. Consideration could be given to placing recommendations into clinical practice guidelines relating to the order in which opioids are prescribed to patients with CNCP, reserving opioids with greater abuse liability for later steps in order to reduce the risk for the development of opioid use disorder.

The mean MME/day among most of our patients on a CSA was below the dose level recommended by the CDC clinical practice guideline for prescribing opioids for chronic pain [7]. This guideline was aimed at primary care clinicians prescribing opioids for chronic pain outside of active cancer treatment, palliative care, and end-of-life care [7]. The CDC guideline recommends that clinicians should “carefully reassess evidence of individual benefits and risks when considering increasing dosage to ≥50 morphine milligram equivalents (MME)/day, and should avoid increasing dosage to ≥90 MME/day or carefully justify a decision to titrate dosage to ≥90 MME/day**.”** In our population, 21.5% were receiving ≥50 MME/day and 9.7% were receiving ≥90 MME/day. The likelihood of opioid abuse among patients differs by dose with estimated ranges from 0.7% with lower doses (≤36 MME/day) to 6.1% with higher doses (≥120 MME/day) as compared to 0.004% in patients not prescribed opioids [7]. CSAs are intended to increase adherence through “contingency contracting” which leverages written documents delineating expected behaviors and the consequences contingent upon these behaviors [32]. To the extent that they incorporate adherence monitoring (e.g., drug evaluation, urine drug screening, and pill counts), CSAs may reduce the risk for dose escalation and the development of opioid use disorders [17] although the data for this is limited [11].

Eighteen percent of our population had contracts terminated by the end of the study period. Twelve percent of our population had CSA discontinuation for violation or patient preference; however, we were not able to ascertain the type of violation warranting discontinuation. Previous studies have observed that 17% of contracts were cancelled by the clinician [19]. The most common reason for this was a urine toxicology screen positive for marijuana or cocaine. Controversy exists regarding the proper corrective action when illicit drugs are discovered. Clinicians may discharge patients from a CSA for this discovery, but some experts recommend preserving the therapeutic alliance with patients and using it as an opportunity to educate or facilitate treatment for other drugs of addiction [33].

Only 6.9% of our population was on a clearly identified tapering plan. We observed a higher mean MME/day among patients with an opioid tapering plan compared to those without a tapering plan with no significant differences in pain scores. The CDC guideline recommends that patients on higher doses of opioids (≥90 MME/day) should be informed of the risks of overdose and offered the opportunity to work toward tapering to safer dosages [7]. Significant barriers to engaging patients in opioid tapering exist including patient perception of low risk for overdose, increased pain with tapering, lack of effectiveness of non opioid pain treatment modalities, and opioid withdrawal [34]. However, available evidence suggests stable or improved pain after an opioid taper [35]. Opioid withdrawal can be avoided through gradual tapering, and the daily dose to prevent acute withdrawal is approximately 25% of the previous day’s dose [36]. Maintaining a healthy therapeutic relationship with CNCP patients can enhance patient care [37] and facilitate tapering if deemed appropriate by the treating clinician [34]. CSAs should be leveraged as an opportunity to engage patients in discussions about the benefits of ongoing opioid use rather than an automatic renewal system and opioid maintenance program. The percentage of patients who should be tapering within a CSA program at any given time is unknown.

The major strength of our study is the size of the population on a CSA for CNCP. A limitation of our study includes our evaluation of a population in a single center in the Midwest through a convenience sampling frame with a low prevalence of minority populations which limits the generalizability to other primary care practices. Another limitation is that we could not ascertain the precise reasons for contract discontinuation due to inconsistent reporting in the EHR, and we did not assess for the prevalence of possible opioid use disorder.

## Conclusions

CSAs have been proposed as contingency contracting, but their greatest strength may lie in providing clinicians an opportunity to take a population health management approach to manage patients with CNCP on opioids. EHR registries that could alert clinicians to opioid doses exceeding pre-determined thresholds, drug screens that are positive for illicit substances, and patients at high risk for opioid use disorder may hold tremendous potential for mitigating risk for patients and providers and improve the overall care of patients on opioids for CNCP.

## Abbreviations

CCI: Charlson Comorbidity Index
CDC: Centers for Disease Control and Prevention
CNCP: Chronic non cancer pain
CSA: Controlled substance agreement
EHR: Electronic health record
GAD-7: Generalized Anxiety Disorder-7 Item Scale
MME: Morphine milligram equivalents
ORT: Opioid Risk Assessment Tool
PHQ-9: Patient Health Questionnaire
SD: Standard deviation

## References

[1] St Sauver JL, Warner DO, Yawn BP (2013). Why patients visit their doctors: assessing the most prevalent conditions in a defined American population. Mayo Clin Proc.

[2] Hardt J, Jacobsen C, Goldberg J, Nickel R, Buchwald D (2008). Prevalence of chronic pain in a representative sample in the United States. Pain Med.

[3] Nahin RL (2015). Estimates of pain prevalence and severity in adults: United States, 2012. J Pain Official J Am Pain Soc..

[4] Reid MC, Engles-Horton LL, Weber MB, Kerns RD, Rogers EL, O'Connor PG (2002). Use of opioid medications for chronic noncancer pain syndromes in primary care. J Gen Intern Med.

[5] Zerzan JT, Morden NE, Soumerai S (2006). Trends and geographic variation of opiate medication use in state Medicaid fee-for-service programs, 1996 to 2002. Med Care.

[6] Martell BA, O'Connor PG, Kerns RD (2007). Systematic review: opioid treatment for chronic back pain: prevalence, efficacy, and association with addiction. Ann Intern Med.

[7] Dowell D, Haegerich TM, Chou R (2016). CDC guideline for prescribing Opioids for chronic pain--United States, 2016. JAMA.

[8] Manchikanti L, Abdi S, Atluri S (2012). American Society of Interventional Pain Physicians (ASIPP) guidelines for responsible opioid prescribing in chronic non-cancer pain: part 2--guidance. Pain Physician..

[9] Chou R, Fanciullo GJ, Fine PG (2009). Clinical guidelines for the use of chronic opioid therapy in chronic noncancer pain. J Pain Official J Am Pain Soc.

[10] Jamison RN, Serraillier J, Michna E (2011). Assessment and treatment of abuse risk in opioid prescribing for chronic pain. Pain Res Treat.

[11] Starrels JL, Becker WC, Alford DP, Kapoor A, Williams AR, Turner BJ (2010). Systematic review: treatment agreements and urine drug testing to reduce opioid misuse in patients with chronic pain. Ann Intern Med.

[12] Arnold RM, Han PK, Seltzer D (2006). Opioid contracts in chronic nonmalignant pain management: objectives and uncertainties. Am J Med.

[13] Collen M (2009). Analysis of controlled substance agreements from private practice physicians. J Pain Palliat Care Pharmacother.

[14] Watkins A, Wasmann S, Dodson L, Hayes M (2004). An evaluation of the care provided to patients prescribed controlled substances for chronic nonmalignant pain at an academic family medicine center. Fam Med.

[15] Penko J, Mattson J, Miaskowski C, Kushel M (2012). Do patients know they are on pain medication agreements? Results from a sample of high-risk patients on chronic opioid therapy. Pain medicine (Malden, Mass).

[16] Khalid L, Liebschutz JM, Xuan Z (2015). Adherence to prescription opioid monitoring guidelines among residents and attending physicians in the primary care setting. Pain Med.

[17] Manchikanti L, Manchukonda R, Damron KS, Brandon D, McManus CD, Cash K (2006). Does adherence monitoring reduce controlled substance abuse in chronic pain patients?. Pain Physician.

[18] Adams NJ, Plane MB, Fleming MF, Mundt MP, Saunders LA, Stauffacher EA (2001). Opioids and the treatment of chronic pain in a primary care sample. J Pain Symptom Manag.

[19] Hariharan J, Lamb GC, Neuner JM (2007). Long-term opioid contract use for chronic pain management in primary care practice. A five year experience. J Gen Intern Med.

[20] Busse JW, Mahmood H, Maqbool B (2015). Characteristics of patients receiving long-term opioid therapy for chronic noncancer pain: a cross-sectional survey of patients attending the pain Management Centre at Hamilton General Hospital, Hamilton, Ontario. CMAJ Open.

[21] Kroenke K, Spitzer RL, Williams JB (2001). The PHQ-9: validity of a brief depression severity measure. J Gen Intern Med.

[22] Spitzer RL, Kroenke K, Williams JB, Lowe B (2006). A brief measure for assessing generalized anxiety disorder: the GAD-7. Arch Intern Med.

[23] Charlson ME, Pompei P, Ales KL, MacKenzie CR (1987). A new method of classifying prognostic comorbidity in longitudinal studies: development and validation. J Chronic Dis.

[24] Deyo RA, Cherkin DC, Ciol MA (1992). Adapting a clinical comorbidity index for use with ICD-9-CM administrative databases. J Clin Epidemiol.

[25] Quan H, Sundararajan V, Halfon P (2005). Coding algorithms for defining comorbidities in ICD-9-CM and ICD-10 administrative data. Med Care.

[26] Von Korff M, Saunders K, Thomas Ray G (2008). De facto long-term opioid therapy for noncancer pain. Clin J Pain.

[27] Washington State Agency Medical Directors’ Group (AMDG) (2015). Interagency Guideline on Prescribing Opioids for Pain.

[28] Brown RT, Zuelsdorff M, Fleming M (2006). Adverse effects and cognitive function among primary care patients taking opioids for chronic nonmalignant pain. J Opioid Manag.

[29] Sullivan MD, Edlund MJ, Zhang L, Unutzer J, Wells KB (2006). Association between mental health disorders, problem drug use, and regular prescription opioid use. Arch Intern Med.

[30] Edlund MJ, Martin BC, Fan MY, Devries A, Braden JB, Sullivan MD (2010). Risks for opioid abuse and dependence among recipients of chronic opioid therapy: results from the TROUP study. Drug Alcohol Depend.

[31] Wightman R, Perrone J, Portelli I, Nelson L (2012). Likeability and abuse liability of commonly prescribed opioids. J Med Toxicol Official J Am College Med Toxicol.

[32] Kirkpatrick AF, Derasari M, Kovacs PL, Lamb BD, Miller R, Reading A (1998). A protocol-contract for opioid use in patients with chronic pain not due to malignancy. J Clin Anesth.

[33] New York State Office of Alcoholism and Substance Abuse Services (2012). Clinical Practice Guidance Number 2012.3: Guidance on Urine Drug Testing.

[34] Frank JW, Levy C, Matlock DD (2016). Patients’ perspectives on tapering of chronic Opioid therapy: a qualitative study. Pain Med.

[35] Berna C, Kulich RJ, Rathmell JP (2015). Tapering long-term Opioid therapy in chronic noncancer pain: evidence and recommendations for everyday practice. Mayo Clin Proc.

[36] Fishbain DA, Rosomoff HL, Cutler R (1993). Opiate detoxification protocols. A clinical manual. Ann Clin Psychiatry.

[37] Upshur CC, Bacigalupe G, Luckmann R (2010). “They don’t want anything to do with you”: patient views of primary care management of chronic pain. Pain Medicine.

